# Overexpression of EZH2 is associated with the poor prognosis in osteosarcoma and function analysis indicates a therapeutic potential

**DOI:** 10.18632/oncotarget.9518

**Published:** 2016-05-20

**Authors:** Ranran Sun, Jacson Shen, Yan Gao, Yubing Zhou, Zujiang Yu, Francis Hornicek, Quancheng Kan, Zhenfeng Duan

**Affiliations:** ^1^ Department of Infectious Diseases, The First Affiliated Hospital of Zhengzhou University, Zhengzhou, Henan, 450052, People's Republic of China; ^2^ Sarcoma Biology Laboratory, Center for Sarcoma and Connective Tissue Oncology, Massachusetts General Hospital, Boston, MA, 02114, USA

**Keywords:** osteosarcoma, EZH2, tissue microarray, proliferation, apoptosis

## Abstract

Osteosarcoma is a primary malignant bone tumor that has a poor prognosis due to local recurrence, metastasis, and chemotherapy resistance. Therefore, there is an urgent need to develop novel potential therapeutic targets for osteosarcoma. Enhancer of zeste homologue 2 (EZH2) is a member of the polycomb group of proteins, which has important functions in epigenetic silencing and cell cycle regulation. Overexpression of EZH2 has been found in several malignancies, however, its expression and the role of EZH2 in osteosarcoma is largely unknown. In this study, we examined EZH2 expression by immunohistochemistry in a large series of osteosarcoma tissues in association with tumor characteristics and patient outcomes. EZH2 expression was also analyzed in a microarray dataset of osteosarcoma. Results showed that higher expression of EZH2 was significantly associated with more aggressive tumor behavior and poor patient outcomes of osteosarcoma. We subsequently investigated the functional and therapeutic relevance of EZH2 as a target in osteosarcoma. Immunohistochemical analysis indicated that EZH2 expression was significantly associated with more aggressive tumor behavior and poorer patient outcomes of osteosarcoma. EZH2 silencing by siRNA inhibited osteosarcoma cell growth, proliferation, migration, and invasion. Moreover, suppression of EZH2 attenuated cancer stem cell functions. Similar results were observed in osteosarcoma cells treated with EZH2 specific inhibitor 3-deazaneplanocin A (DZNep), which exhausted cellular levels of EZH2. These results suggest that EZH2 is critical for the growth and metastasis of osteosarcoma, and an epigenetic therapy that pharmacologically targets EZH2 via specific inhibitors may constitute a novel approach to the treatment of osteosarcoma.

## INTRODUCTION

Osteosarcoma is the most common primary malignant tumor of bone in young adults and adolescents. Although the standard treatment of surgical resection and adjuvant chemotherapy has significantly improved the five-year survival rate of osteosarcoma patients to approximately 60–70%, there has been no impressive progress in improving the survival rate of those with recurrence or metastasis over the last three decades [1–3]. Unfortunately, most of the current strategies have limited efficacy in the treatment of metastatic and recurrent osteosarcoma, which remains a major challenge in bone cancer fields. Therefore, there is an urgent need to develop novel early molecular markers of diagnostic and therapeutic targets for osteosarcoma. Meanwhile, the underlying mechanism of osteosarcoma cell growth and proliferation remains largely unclear, which needs to be further elucidated.

As a member of the polycomb group protein (PcG) family and the core enzymatically active component of the polycomb repressive complex 2 (PRC2), Enhancer of zeste homologue 2 (EZH2) is believed to function by catalyzing histone H3 trimethylation at lysine 27 (H3K27me3) in targeted gene promoters to repress gene expression [4, 5]. EZH2 is also required for the stable transmission of gene expression patterns to progeny cells throughout development [6]. Accumulating evidence indicates that EZH2 is involved in fundamental cellular processes, such as cell proliferation and differentiation, cell cycle regulation and fate decision, carcinogenesis, cancer stem cell maintenance, and drug resistance [7–11]. Therefore, it is not surprising that EZH2 has received boosted attention in the oncology field in recent years. There is growing evidence that EZH2 can be regulated at transcriptional and post-translational levels in cancers [12, 13]. Limited expression of EZH2 was found in healthy tissues, while increased levels of EZH2 have been widely observed during tumorigenesis and progression [13]. Furthermore, it has been well confirmed that EZH2 is extensively overexpressed and is associated with more aggressive behavior and poor prognosis in a wide range of cancer types, including prostate cancer, breast cancer, melanoma, and non-small cell lung carcinoma [14–18]. Knockdown of EZH2 results in reduced proliferation, increased apoptosis, and diminished tumorigenicity in cancer cells [19–21]. However, the expression status and functional role of EZH2 in tumorigenesis and progression of osteosarcoma has not yet been fully investigated.

Recently, it has been confirmed that a cyclopentenyl analog of 3-deazaadenosine, 3-deazaneplanocin A (DZNep), can reduce EZH2 expression in breast cancer cells and cause concomitant inhibition of H3K27me3 expression, which causes derepression of epigenetically silenced target genes [22]. Moreover, DZNep inhibits proliferation and promotes apoptosis in several types of cancer cells [23–26]. These findings indicate that pharmacologically targeting EZH2 via DZNep may be an appropriate therapeutic approach to cancer treatment. Currently, however, no studies on the activity of DZNep in osteosarcoma cells have been reported.

To this end, we first examined the extent of EZH2 expression in osteosarcoma cell lines and tumor samples in a 64 osteosarcoma patient cohort via tissue microarray (TMA) technology, and then analyzed the relationship between EZH2 expression and clinical behavior of osteosarcoma. We further mechanistically investigated the functional role of EZH2 in the regulation of cell proliferation, apoptosis, clonogenicity, cancer stem cell maintenance, cell migration, and invasive malignant behaviors *in vitro*. Finally, we further assessed the effects of DZNep on EZH2 protein expression in osteosarcoma cells.

## RESULTS

### EZH2 expression and association with clinicopathologic features

**Figure 1:**
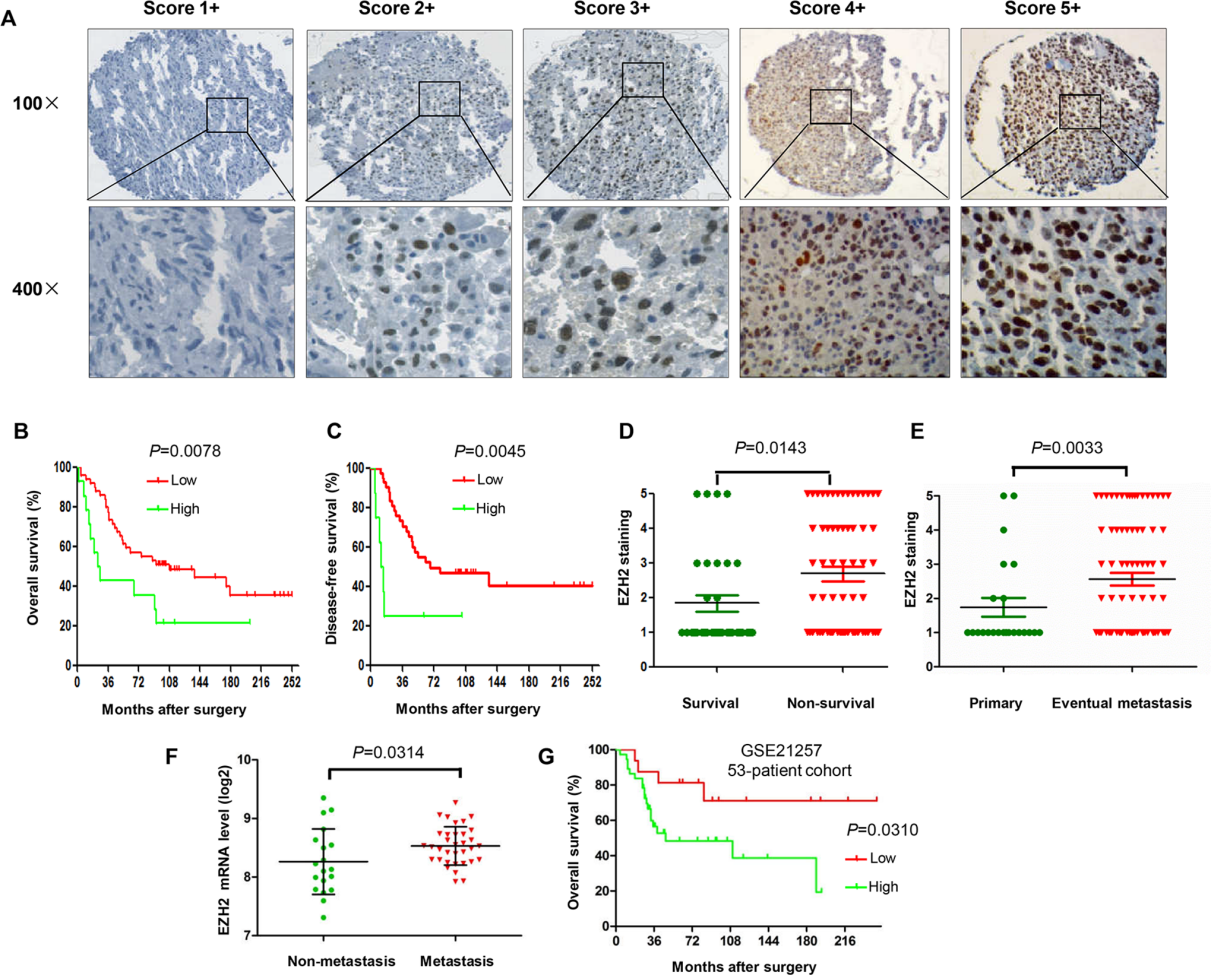
Association of EZH2 protein expression and clinical outcome in osteosarcoma patients (**A**) Representation images of different immunohistochemical staining intensities of EZH2 protein expression in osteosarcoma tissues are shown on TMA sections. EZH2 immunoreactivity was found mostly in the nucleus of tumor cells. EZH2 expression was classified into 1 to 5 categories according to the EZH2 staining intensity and extension values: 1+ (score 0), 2+ (score 1–2), 3+ (score 3–4), 4+ (score 6–8), and 5+ (score 9–12) staining. For statistical analysis, groups 4+ and 5+ were defined as high expression of EZH2, and the other final scores were considered as low expression. (**B**) Kaplan–Meier curves depicting overall survival rates in the two sets of osteosarcoma patients by EZH2 staining (low and high). (**C**) Kaplan–Meier curves depicting disease free survival rates in the 2 sets of osteosarcoma patients by EZH2 staining (low and high). (**D**) Distribution of EZH2 staining scores among the survival and non-survival patients. (**E**) Distribution of EZH2 staining scores among primary and metastasis patients at initial diagnosis. (**F**) Levels of EZH2 mRNA expression among osteosarcoma patients who developed metastases and patients with non-metastases within five years. (**G**) Kaplan-Meier curves depicting overall survival rates in the two sets of osteosarcoma patients by EZH2 mRNA expression (low and high).

In this study, we performed immunohistochemistry to evaluate the significance of EZH2 expression in tumor tissues from 64 osteosarcoma patients. The immunohistochemistry staining results showed that EZH2 protein expression is mainly detected in the nucleus of osteosarcoma cells (Figure 1A). To investigate the clinical relevance between EZH2 expression and osteosarcoma clinical behavior, we evaluated the clinicopathologic features of the osteosarcoma tumor samples. The correlations between EZH2 expression and clinicopathologic characteristics of tumors are shown in Table 1. In the median 81.5-month follow-up evaluation of the 64 post-operative patients, we found that the expression of EZH2 was significantly associated with post-operative clinical outcomes. By Kaplan-Meier analysis, EZH2-high expression patients had significantly poorer disease free survival (DFS) and overall survival (OS) than EZH2-low expression patients (Figure 1B, 1C). In the subgroup analysis, the mean EZH2 expression for the non-survival group was significantly higher than the survival group for osteosarcoma (*P* = 0.00143; Figure 1D). Moreover, there was a significant association between high nuclear EZH2 expression and metastasis at initial diagnosis (*P* = 0.0299; Figure 1E).

**Table 1:** The relationship between EZH2 expression and clinicopathological features of osteosarcoma

	Clinicopathological features	No. of cases (%)	EZH2 expression High (*n* = 16)	EZH2 expression Low (*n* = 48)	*P*	Survival (%)	Univariate *P*
Age (years)	≤ 32	35 (54.7%)	7 (43.8%)	28 (58.3%)	0.310	52.0%	0.773
	> 32	29 (45.3%)	9 (56.3%)	20 (41.7%)		48.0%	
Gender	Male	40 (62.5%)	8 (50%)	32 (66.7%)	0.250	56.0%	0.588
	Female	24 (37.5%)	8 (50%)	16 (33.3%)		44.0%	
Tumor site	Femur	32 (50%)	9 (56.3%)	23 (47.9%)	0.908	36.0%	0.219
	Tibia	10 (15.6%)	2 (12.5%)	8 (16.7%)		28.0%	
	Humeral bone	7 (23.4%)	2 (12.5%)	5 (10.4%)		12.0%	
	Other	15 (23.4%)	3 (18.8%)	12 (25%)		24.0%	
Metastasis	Absent	19 (29.7%)	1 (5.3%)	18 (33.3%)	0.025	64%	< 0.001
	Present	45 (70.3%)	15 (94.7%)	30 (66.7%)		36%	
Recurrence	Absent	44 (68.8%)	11 (68.8%)	33 (68.8%)	1.000	80.0%	0.189
	Present	20 (31.3%)	5 (31.3%)	15 (31.3%)		20.0%	
Response to preoperative chemotherapy	Good	10 (15.6%)	3 (18.8%)	7 (14.6%)	0.685	24.0%	0.324
	Poor	30 (46.9%)	6 (37.5%)	24 (50%)		36.0%	
	N/A	24 (37.5%)	7 (43.8%)	17 (35.4%)		40.0%	
EZH2 expression	High		16 (25.0%)			88.0%	0.009
	Low			48(75.0%)		12.0%	

To further investigate the clinical relevance between EZH2 expression and osteosarcoma behavior, we also analyzed the relationship between EZH2 mRNA expression and the survival of osteosarcoma patients from 53 osteosarcoma tumor samples in a microarray dataset (GSE21257). Notably, the mRNA expression level of EZH2 was significantly up-regulated in osteosarcoma patients who developed metastases within five years than in patients who did not develop metastases within five years (*P* = 0.0314; Figure 1F). Furthermore, Kaplan-Meier survival analysis revealed that patients with high EZH2 expression had shorter survival in this cohort of osteosarcoma patients (*P* = 0.0310; Figure 1G).

These data demonstrate that EZH2 was overexpressed in primary tumors and increased in the metastatic tumors of patients with osteosarcoma. Furthermore, patients with osteosarcoma presenting with high EZH2 expression exhibited a worse prognosis than those with low EZH2 expression. Thus, monitoring the expression level of EZH2 protein in osteosarcoma specimens may provide additional prognostic information, which is not discernible with current clinical and pathology parameters alone.

### EZH2 is overexpressed in osteosarcoma cell lines and tissues

**Figure 2:**
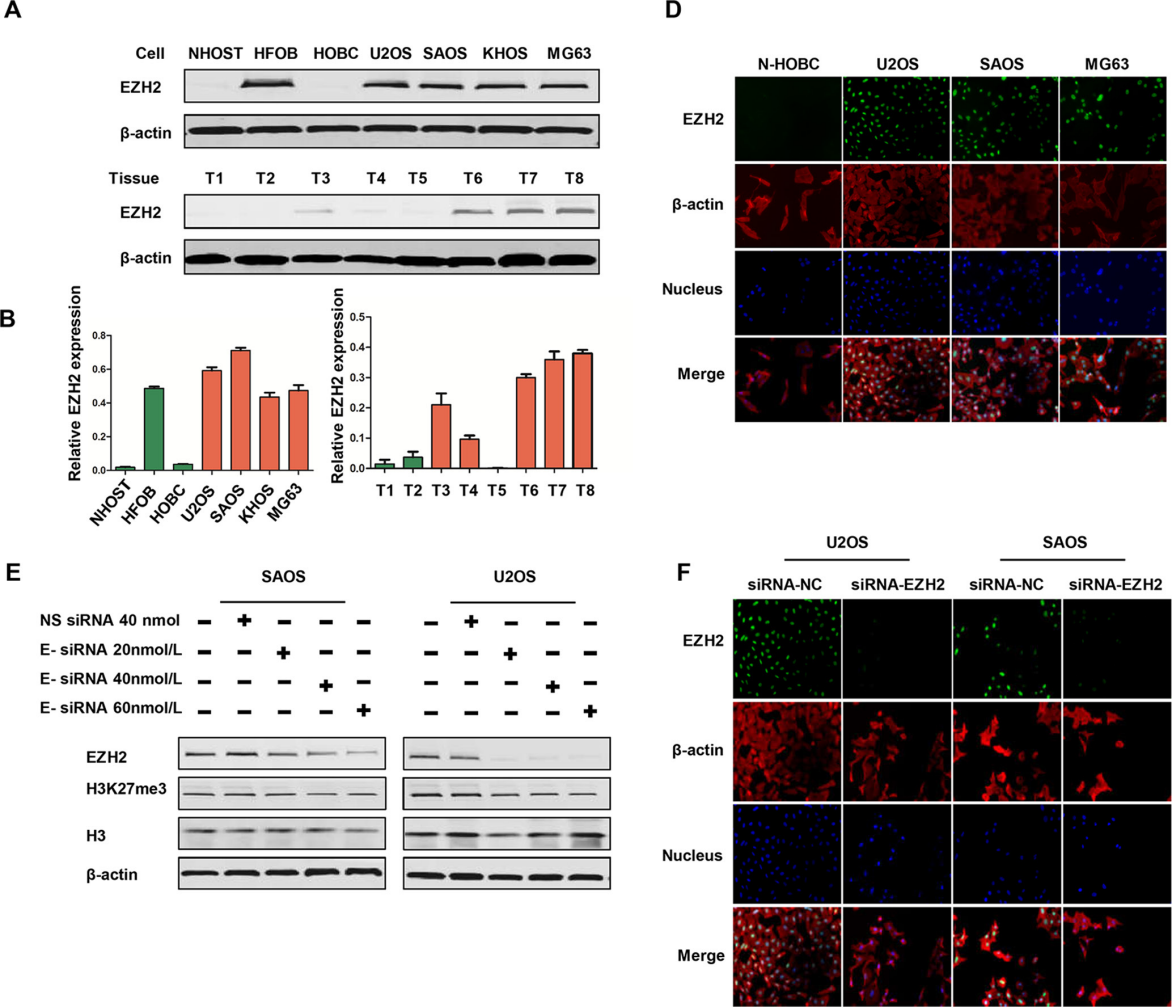
Expression of EZH2 in osteosarcoma cell lines and tissues, and effects of EZH2 knockdown by siRNA in osteosarcoma cell lines (**A**) Expressions of EZH2 in osteosarcoma cell lines (U2OS, SAOS, KHOS, MG63), osteoblast cell lines (NHOST, HFOB, HOBC), and osteosarcoma tissues. (**B** and **C**) Western blots from A were analyzed by densitometry as described in Materials and Methods. Quantitative results of EZH2 expression for cell lines and tissues were presented as relative expression. (**D**) Confirmation of EZH2 expression in osteosarcoma cell lines by immunofluorescence with antibodies to EZH2 (green) and β-actin (red). Hoechst 33342 was added to counterstain the cell nucleus (blue). Green fluorescence of EZH2 protein was mainly localized in the nucleus of osteosarcoma cells. (**E**) siRNA knocked down EZH2 expression in osteosarcoma cells in a dose-dependent manner. (**F**) Confirmation of EZH2 knockdown in osteosarcoma cell lines by immunofluorescence.

To investigate the level of EZH2 expression in osteosarcoma, we detected its protein levels in a series of osteosarcoma cell lines and clinical specimens. Western blot revealed constitutive EZH2 expression in all four osteosarcoma cell lines (KHOS, U2OS, SAOS, and MG63) examined; whereas there was no expression in osteoblast cells (NHOST, HOBC) (Figure 2A, 2B). Expression of EZH2 was also found in osteoblast cell line HFOB, which was established and immortalized by transfection with SV40 large T antigen [27]. To corroborate the cell line data with primary tumor tissues, EZH2 expression was also evaluated in eight osteosarcoma tissues. Different levels of EZH2 expression were observed in these osteosarcoma patient samples (Figure 2A, 2C).

In addition, we performed immunofluorescence to determine subcellular localization in osteosarcoma cell lines. As shown in Figure 2D, immunoflurescent staining showed that EZH2 protein is also localized in the nucleus of osteosarcoma cells, which is consistent with the immunohistochemistry staining of EZH2 in osteosarcoma tissues.

### EZH2 silencing inhibits osteosarcoma cell proliferation, migration, invasion, and clonogenicity activity

Expression of EZH2 has been found to be associated with poor prognosis and increased in metastasis in different cancers as reported by previous studies. To investigate the functional role of EZH2 in osteosarcoma, we used synthetic RNA interference (RNAi) to disrupt EZH2 expression in two osteosarcoma cells lines *in vitro*. U2OS and SAOS cells were transfected with EZH2 siRNA in gradient concentrations, and downregulated expression of EZH2 protein was observed via western blot analysis (Figure 2E). EZH2 immunofluorescence analysis was performed to further confirm the decreased expression of EZH2 after transfection with EZH2 siRNA. Consistent with the results of the osteosarcoma TMA, EZH2 protein expression was mainly localized in the nucleus of osteosarcoma cells. Furthermore, compared with cells transfected with non-specific siRNA, the results of the EZH2 immunofluorescence assay further confirmed the significantly downregulated expression level of EZH2 after transfection with EZH2 siRNA (Figure 2F).

**Figure 3:**
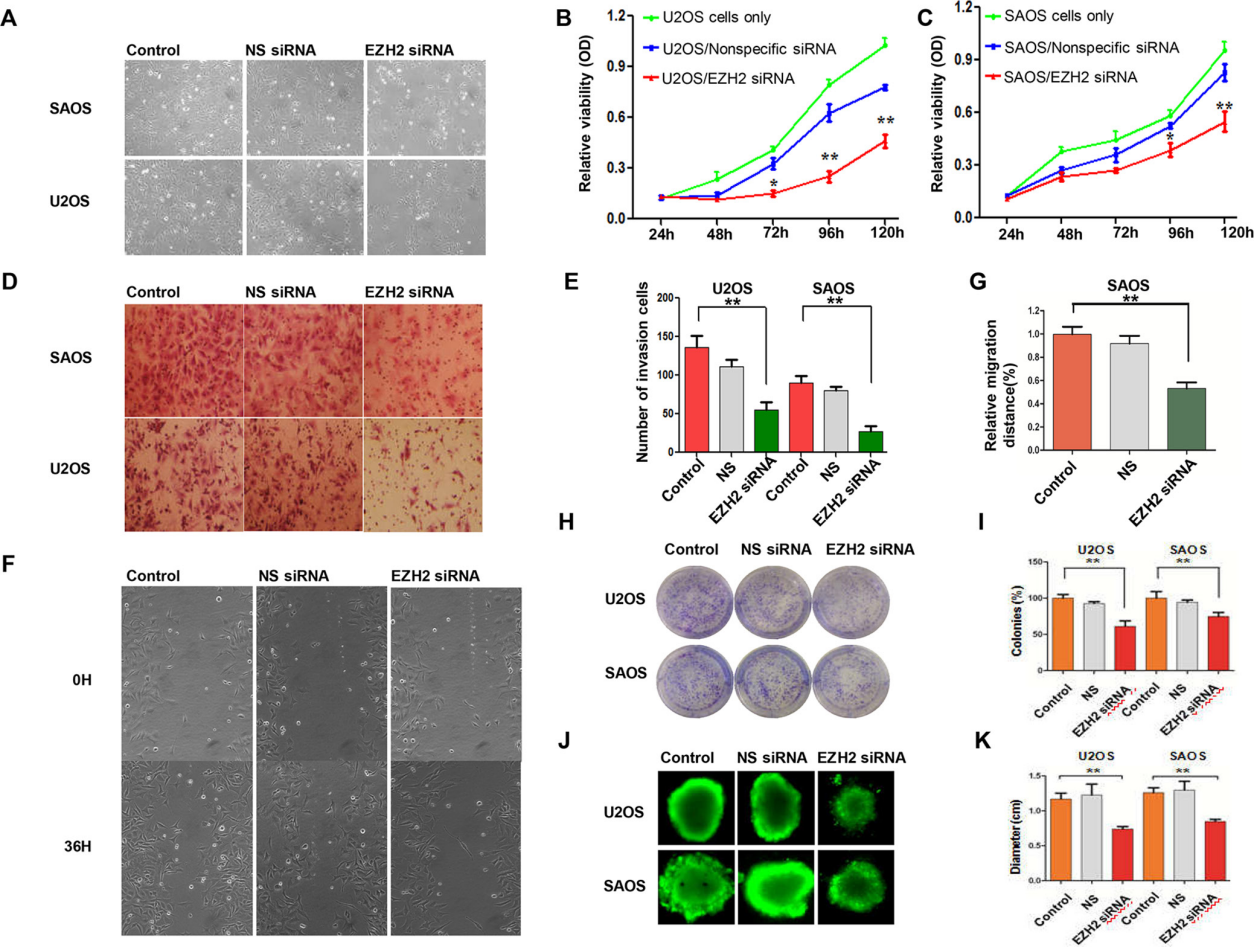
Effects of EZH2 silencing on osteosarcoma cell proliferation, migration, and invasion (**A**) Representative images of osteosarcoma cells after transfection with non-specific siRNA or EZH2 siRNA in a 12-well plate. (**B** and **C**) Effects of EZH2 knockdown on osteosarcoma cell growth and proliferation after transfection with either non-specific siRNA or EZH2 siRNA (*P < 0.01. **P < 0.001). (**D** and **E**) Transwell assays to assess osteosarcoma cell migration after EZH2 knockdown (crystal violet staining of migratory cells; **P < 0.001; scale bar, 50 mm). (**F**) Micrographs of osteosarcoma SAOS cells at 0 and 36 hours after wounding. (**G**) Migration distance of SAOS for each time point and condition (**P < 0.001). (**H** and **I**) Representative results of colony formation of osteosarcoma cell lines transfected with non-specific siRNA or EZH2 siRNA (*P < 0.01. **P < 0.001). (**J** and **K**) Formation of spheres from osteosarcoma cell lines transfected with non-specific siRNA or EZH2 siRNA accessed by three-dimensional cell culture. All the results were reproducible in three independent experiments. The data are presented as the mean ± SD. *P < 0.01).

Since the overexpression of EZH2 is associated with more aggressive tumor behavior and poorer patient outcomes in osteosarcoma, we performed a proliferation assay to determine whether EZH2 silencing could inhibit cell growth. Inhibition of cell growth in SAOS cells was observed 96 h after transfection with EZH2 siRNA (Figure 3A). The MTT assay demonstrated that EZH2 silencing significantly inhibited cell growth after 72 h (*P* < 0.05), while cells transfected with non-specific siRNA revealed no inhibition of growth along with the control group (Figure 3B, 3C). These results confirmed that EZH2 was critical for osteosarcoma cell growth. Moreover, RNAi mediated EZH2 knockout induced growth inhibition in a time-dependent manner in both osteosarcoma cell lines U2OS and SAOS.

To determine the migratory response of osteosarcoma cells to EZH2 knockdown, using a transwell assay, the average number of invasive cells was observed to be reduced approximately 50% and 67% after transfection with EZH2 siRNA as compared with U2OS and SAOS cells transfected with non-specific siRNA (NS) (*P* < 0.001 and *P* < 0.001, respectively) (Figure 3D, 3E). The wound healing assay was also carried out to validate the effects of EZH2 silencing on osteosarcoma cell migration. During the 36 h incubation, compared with the cells transfected with non-specific siRNA, the migration distance of U2OS cells transfected with EZH2 siRNA was remarkably decreased. Similar results were observed in the SAOS cell line (Figure 3F, 3G).

**Figure 4:**
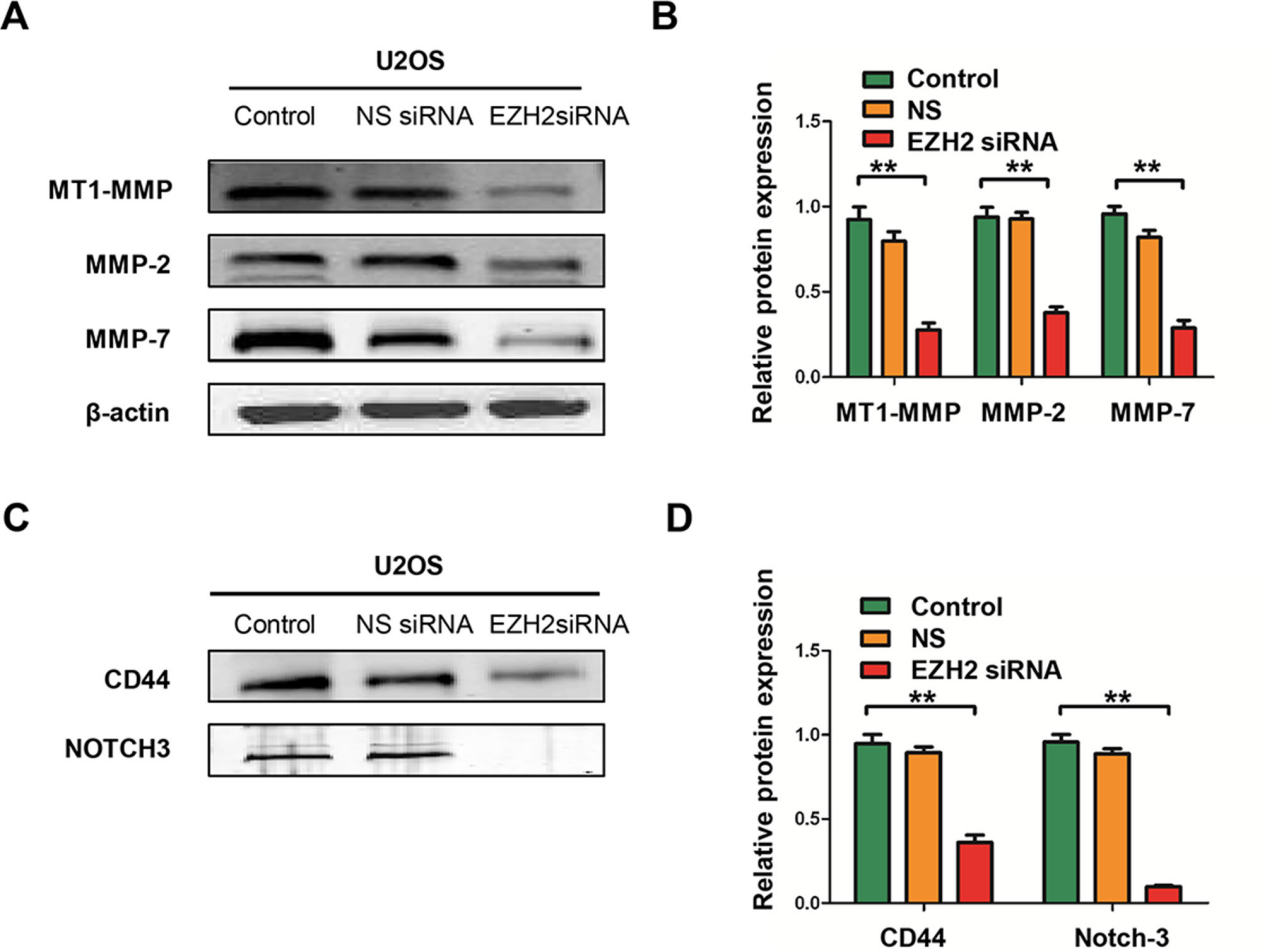
Effects of EZH2 silencing on the expression of metastasis-associated proteins and stem cell markers in osteosarcoma cell lines (**A**) The levels of MT1-MMP, MMP-2, and MMP-7 were detected in cells after transfection with non-specific siRNA or EZH2 siRNA and determined by western blot analysis. (**B**) Western blots from A were analyzed by densitometry, which was carried out using Image J software and normalized to β-actin expression. (**C**) The levels of CD44 and Notch-3 were detected in cells after transfection with non-specific siRNA or EZH2 siRNA and evaluated by western blot analysis. (**D**) Western blots from C were analyzed by densitometry as described in Materials and Methods. All the results were reproducible in three independent experiments. The data are presented as the mean ± SD with **P < 0.001.

Subsequently, we performed the colony formation assay to investigate the effect of EZH2 silencing on clonogenicity and found that the average number of colonies formed by U2OS-EZH2-siRNA and SAOS-EZH2-siRNA cells were significantly lower than the number formed by U2OS-NS and SAOS-NS cells (*P* < 0.001 and *P* < 0.001, respectively), and the sizes of the colonies were also reduced (Figure 3H, 3I). To exclude the possibility of artifact induced by *in vitro* culture, we further confirmed the above results in a mimic *in vivo* environment using a three-dimensional cell culture assay. Consistent with the results obtained from the two-dimensional colony formation assay, the average diameter of cancer spheroids formed from U2OS-EZH2-siRNA and SAOS-EZH2-siRNA cells was significantly lower than the diameter formed from U2OS-NS and SAOS-NS in the three-dimensional cell culture environment (Figure 3J, 3K). These data demonstrate that osteosarcoma cell invasive and migratory activities were significantly suppressed after EZH2 silencing. Meanwhile, silencing EZH2 inhibited the expression of metastasis-associated proteins MT1-MMP, MMP-2, and MMP-7, indicative of reduced invasive potential (Figure 4A, 4B).

### Effect of EZH2 knockdown on the expression of CD44 and Notch-3

Previous studies have shown that EZH2 also plays a role in cancer stem cell via regulation of stem cell gene expression. Expression of EZH2 is required for maintenance of a stem cell state in different cancers, including prostate cancer, breast cancer, and glioblastoma [11, 28, 29]. We therefore examined the effect of EZH2 silencing on the protein expressions of cancer stem cell markers; include CD44 and Notch-3 in osteosarcoma cells. Figure 4C and Figure 4D show that the expressions of CD44 and Notch-3 were remarkably decreased in U2OS cells transfected with EZH2 siRNA. These results indicate that EZH2 silencing may also attenuate cancer stem function in osteosarcoma.

### Effect of EZH2 knockdown on apoptosis in osteosarcoma cell lines

**Figure 5:**
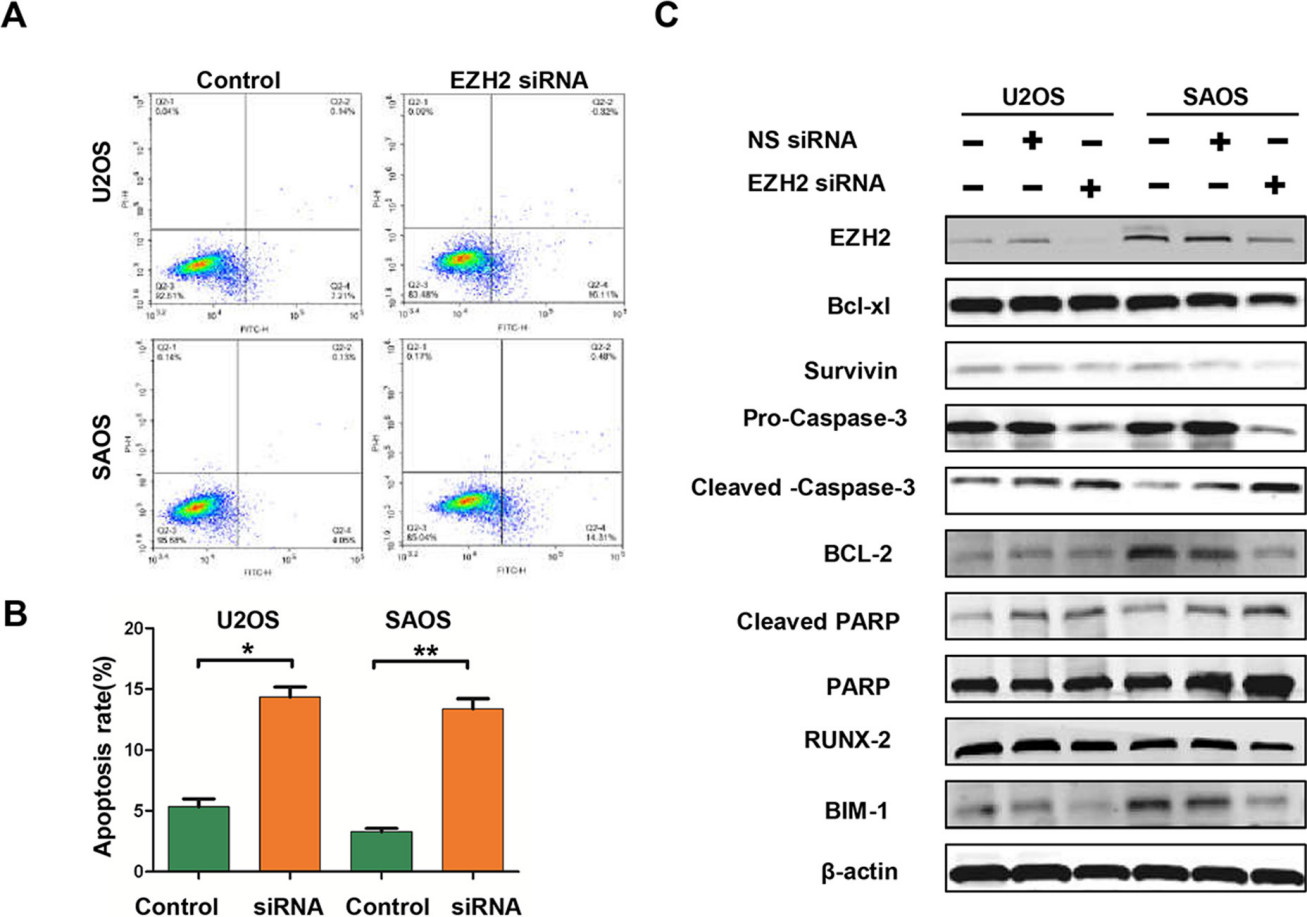
Effects of EZH2 silencing on apoptosis in osteosarcoma cells (**A** and **B**) Apoptosis was determined by cytometric analysis. Cells stained with annexin-V-APC were considered as apoptotic. The apoptotic index was defined as the percentage of apoptotic cells (*P < 0.01). (**C**) The levels of Bcl-xl, surviving, caspase-3, Bcl-2, PARP, BIM-1, and RUNX-2 were detected in osteosarcoma cells after transfection with non-specific siRNA or EZH2 siRNA and determined by western blot analysis.

To investigate the underlying mechanisms of inhibition in growth and survival by EZH2 silencing, we performed flow cytometry analysis and apoptosis-associated protein measurement. As demonstrated by flow cytometry analysis, significantly increased apoptosis rates were observed in EZH2 silenced cells (Figure 5A, 5B). Meanwhile, EZH2 knockdown decreased the expressions of anti-apoptotic proteins, including survivin, Bcl-xl, Bcl- 2, Bim-1, and Pro-caspase-3 (Figure 5C). We also observed an increase in the pro-apoptotic cleaved-caspase-3 and cleaved-PARP proteins in cells transfected with the EZH2 siRNA (Figure 5C). Quantitative measurement of these apoptotic associated proteins confirmed the significant effect of EZH2 knockdown on apoptosis in U2OS and SAOS cells (data were not shown). Thus, these results indicated that EZH2 silencing could induce cell apoptosis *via* regulation of apoptotic proteins in osteosarcoma cells.

### DZNep inhibits EZH2 expression and cell proliferation in osteosarcoma cell lines

**Figure 6:**
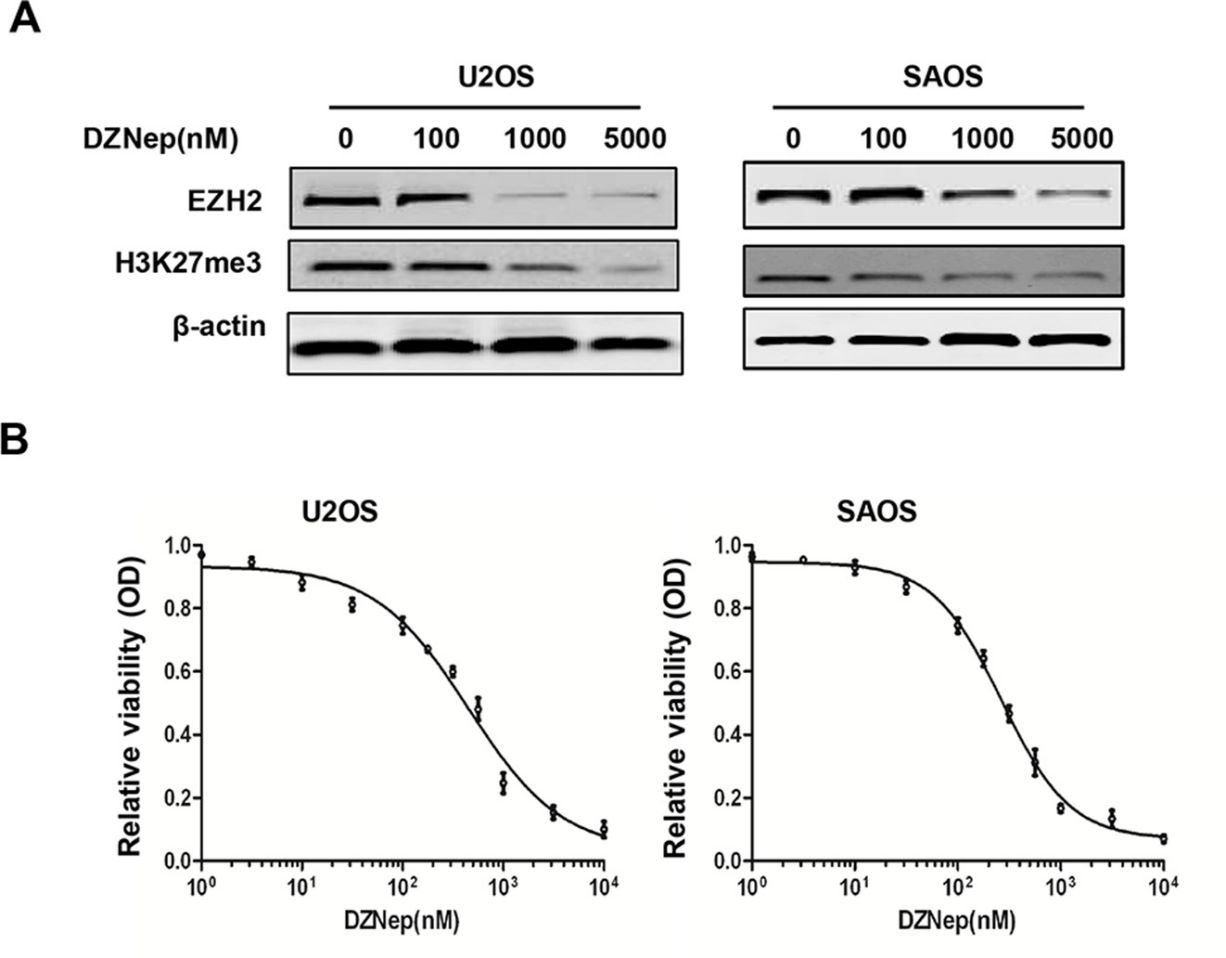
Effects of EZH2 inhibitor DZNep on the expression of EZH2 and cell growth in osteosarcoma cell lines (**A**) DZNep decreased the expression of EZH2 and H3K27me3 in osteosarcoma cells in a dose-dependent manner. (**B**) DZNep inhibited osteosarcoma cell proliferation. Cells were treated with DZNep at the indicated concentrations. The relative sensitivity of each line to DZNep was determined by the MTT assay.

EZH2 inhibitor DZNep has been shown to induce apoptosis in different cancer cells. Consistent with the findings of EZH2 siRNA silencing in osteosarcoma cells in this study, treatment of osteosarcoma cell lines U2OS and SAOS with DZNep resulted in significant reduction of EZH2 protein levels and subsequently decrease H3K27me3 levels in a dose-dependent manner (Figure 6A). Furthermore, we evaluated the effect of DZNep treatment on the growth of osteosarcoma cells by growth inhibition assay. Significant proliferation inhibition was observed when osteosarcoma cell lines were treated with DZNep (Figure 6B). The IC50 values for DZNep in the osteosarcoma cells lines were 0.45 μM (U2OS) and 0.26 μM (SAOS). These results indicate the potential therapeutic use of DZNep in osteosarcoma treatment.

## DISCUSSION

In the present study, we found that EZH2 was constitutively highly expressed in osteosarcoma cell lines and tissues. Further correlation analyses revealed that abnormally high expression of EZH2 was positively correlated with increased aggressive and metastatic behavior, indicating that EZH2 expression may contribute to the progression of osteosarcoma by functioning as an oncogene. These data are consistent with previous results demonstrating overexpression of EZH2 in other types of cancer, including prostate, breast, ovarian, and pancreatic cancers, as well as the findings that overexpression of EZH2 is associated with aggressive and metastatic behavior and poor prognosis in cancers [18, 30, 31]. By Kaplan-Meier analysis, we present the first study to examine the prognostic value of EZH2 expression in osteosarcoma patients using high-throughput tissue microarray analysis. The results confirmed that higher EZH2 expression was associated with a poorer prognosis in osteosarcoma. Multivariate analysis further corroborated that EZH2 expression was an independent prognostic factor of outcome. These results suggest that EZH2 may play an important role in osteosarcoma development. Dysregulated expression of EZH2 may be involved in the progression of osteosarcoma, and may be a valuable prognostic marker that distinguishes less aggressive osteosarcoma from those at risk of lethal progression.

To examine the role of EZH2 in osteosarcoma cell growth and proliferation, we used RNAi to disrupt EZH2 expression in osteosarcoma cells *in vitro*. Our study observed that knockdown of EZH2 significantly inhibited osteosarcoma cell growth, survival, and clonogenicity, as well as decreased cell migration and invasion ability, implying that EZH2 plays an important role in osteosarcoma progression. In order to further investigate the function of EZH2 in osteosarcoma, we mimicked the *in vivo* environment by using a three-dimensional cell culture. Knockdown of EZH2 expression in osteosarcoma cells resulted in slower migration and repressed invasion activity. Consistent with our findings in the tissue microarray analysis that the presence of EZH2 in osteosarcoma is linked to higher metastatic migration, similar observations have also been reported in pancreatic cancer, prostate cancer, oral tongue squamous cell carcinoma, and breast cancer [32–34]. Metastasis is a complex biological process in which tumor cells acquire the invasive and migration abilities for disseminating from a primary tumor to distant secondary organs or tissues [22, 23]. We found reduced expressions of MT1-MMP, MMP-2, and MMP-7 after EZH2 knockdown in osteosarcoma cells. These proteins have been shown to play critical roles in the processes of tumor cell invasion and metastasis by pericellular extracellular matrix (ECM) degradation in osteosarcoma [35, 36]. Furthermore, stem cell markers of CD44 and Notch-3 were decreased in osteosarcoma cells after inhibition of EZH2. These results indicate that EZH2 silencing may also attenuate cancer stem function in osteosarcoma. Other stem cell markers, including CD133, Ki-67, and ABCG2 have been used to sort osteosarcoma stem cells [37, 38]. All of these results are indicative of the critical role of EZH2 in cancer cell proliferation, migration, and invasion. Interestingly, while our manuscript was in the preparation, a study reported that overexpression of EZH2 is associated with poor prognosis in osteosarcoma. In addition, this study also showed that silencing EZH2 resulted in tumor growth inhibition, apoptosis, and chemosensitivity enhancement. Moreover, suppression of EZH2 markedly inhibited tumor growth and lung metastasis *in vivo* [39].

Inhibition of apoptosis is one of the major mechanisms in cancer development and ultimately leads to the expansion of neoplastic cells with deregulated proliferation and accumulation of genetic instability and mutations. Knockdown EZH2 has been shown to induce apoptosis in different cancers [12, 15, 33]. However, the mechanisms of apoptosis induction by EZH2 inhibition remain poorly understood. To further elucidate the molecular mechanisms underlying the effects of EZH2 in osteosarcoma cells, we investigated several apoptotic related proteins after EZH2 silencing and the results showed that several anti-apoptotic proteins were reduced, as well as activation of caspase-3 and PARP cleavage. Our findings indicate that EZH2 functions as a key coordinator involved in apoptosis signaling in osteosarcoma. Furthermore, we confirmed for the first time that DZNep is effective in reducing EZH2 expression and inhibits tumor growth in osteosarcoma. Numerous studies suggest that DZNep induces death in tumor cells through EZH2 downregulation and H3K27me3 reduction [12, 13, 25]. In our current osteosarcoma model, we also found that DZNep reduces EZH2 at the protein level and subsequently decreases H3K27me3. The data showed that pharmacologic disruption of EZH2 via DZNep inhibited growth in osteosarcoma cell lines in a dose-dependent manner. These findings were consistent with findings from previous studies in different types of tumors.

In summary, we have provided evidence demonstrating that elevated EZH2 expression in osteosarcoma is significantly associated with tumor metastasis and poor prognosis. Measuring EZH2 expression level may carry potential as a novel molecular biomarker of osteosarcoma. In addition, we have shown that EZH2 plays a crucial role in mediating cell proliferation, invasion, and migration. EZH2 inhibitor DZNep can inhibit the growth of osteosarcoma cell lines, indicating that pharmacologically targeting EZH2 via an inhibitor may constitute a novel approach to the treatment of osteosarcoma.

## MATERIALS AND METHODS

### Patients, specimens, and cell lines

This study was approved by the ethics committee of Massachusetts General Hospital, and all patients provided informed consent. This study was conducted with the approval of the MGH Institutional Review Board (IRB protocol #:2007-P-002464/5). Cancer tissue specimens were obtained from 64 osteosarcoma patients between 1993 and 2009 at the Department of Orthopaedic Surgery, Massachusetts General Hospital, Boston, USA. Haematoxylin and eosin-stained (HE) slides from each tissue block were read by a pathologist to obtain representative triplicate 0.5-mm-diameter core biopsies. Clinical information of all the patients was collected and managed from the archives, including age, gender, tumor location, tumor stage (primary/metastasis/recurrent), whether the patient received pre-operative chemotherapy or not, the disease status, and the follow-up months. Metastasis is defined as the presence of metastatic disease in the initial diagnosis. Recurrence is defined as the presence of recurrence in the last follow-up visit of the patient. The tissue specimens were fixed in 4% formalin and embedded in paraffin for immunohistochemical staining. To determine the immunohistochemical expression of EZH2 in osteosarcoma, we used Formalin-Fixed, Paraffin-Embedded (FFPE) tumors tissue placed in a tissue microarray (TMA). Representative areas of osteosarcoma tumor specimens from the paraffin-embedded samples for each case were selected and circled as previously described [40, 41]. Briefly, in order to ensure accurate representation of the selected cores, three areas of tumor parts per case were selected for assembling the recipient master block. The TMA was constructed by the Tissue Microarray and Imaging Core at the Dana-Farber/Harvard Cancer Center.

The human osteoblast cell line HOB-c was purchased from PromoCell GmbH (Heidelberg, Germany), and the Simian Vacuolating Virus 40 (SV40) large T antigen transfected and immortalized human osteoblast HFOB was purchased from ATCC (Manassas, VA). The human osteosarcoma cell lines U2OS, SAOS, and MG63 were also purchased from ATCC. The human osteosarcoma cell line KHOS was provided by Dr. Efstathios Gonos (Institute of Biological Research and Biotechnology, Athens, Greece). Cells lines were maintained in RPMI 1640 supplemented with 1% penicillin/streptomycin and 10% fetal bovine serum (FBS) in a 5% CO_2_, 37°C cell culture incubator.

### Expression data sets

A set of microarray data (GSE21257) that included gene expression from a total of 53 patient osteosarcoma tissues was downloaded from the Gene Expression Omnibus (GEO) (http://www.ncbi.nlm.nih.gov/geo) [42]. Expression results of EZH2 from this data set were retrieved, and the association between the expression levels of EZH2 and the clinical significance were analyzed using BRB-array tools [43].

### Immunohistochemical staining and evaluation of EZH2 expression

Briefly, 5-μm-thick TMA sections were deparaffinized and hydrated as previously described [40, 41], and antigen retrieval was performed with Target Retrieval Solution (Dako, Carpinteria, CA) following the manufacturer's instructions. Peroxide blocking was performed at room temperature for 30 minutes with 3% H_2_O_2_ in methanol. Slides were then blocked with goat serum, avidin solution, and biotin solution. The slides were incubated with EZH2 primary antibody (Monoclonal, catalogue number:5246, Cell Signaling Technology, MA) at 4°C overnight (1:100 dilution, in 1% bovine serum albumin PBS) and then probed with biotinylated goat anti-rabbit secondary antibody (Vector Laboratories, Burlingame, CA) and high-sensitivity streptavidin–HRP conjugate. To visualize staining, slides were incubated in 3, 30-diaminobenzidine in 0.1% H_2_O_2_ in Tris–HCl buffer, and subsequently counterstained with Hematoxylin QS (Vector Laboratories). We used synovial sarcoma tissues as external positive controls for EZH2 staining, and goat serum (without EZH2 primary antibody) as the negative controls for EZH2 staining.

Sections were semi-quantitatively scored for the EZH2 staining patterns as follows: the staining extent in each core was scored as 1+ (< 25% staining of tumor cells), 2+ (25–50% staining of tumor cells), 3+ (50% to 75% staining of tumor cells), or 4+ (> 75% staining of tumor cells). Additionally, the staining intensity was quantified as 0 (negative), 1+ (weak), 2+ (intermediate), or 3+ (strong). The final immunoreaction score was obtained by multiplying the intensity and extension values (range 0–12) and the samples were grouped as 1+ (score 0), 2+ (score 1–2), 3+ (score 3–4), 4+ (score 6–8), and 5+ (score 9–12) staining. Meanwhile, for statistical purposes, scores of 4+ and 5+ were defined as high expression and the other final scores were considered as low expression. Categorizing the EZH2 staining was completed by two independent investigators (Ranran Sun and Yan Gao) in the Sarcoma Research Laboratory with an approach as previously described [40], who were blinded to the clinicopathologic data, with a consensus reached in all cases. EZH2 staining images were obtained using a Nikon Eclipse Ti-U fluorescence microscope (Nikon Instruments, Inc., Japan) with a SPOT RT digital camera (Diagnostic Instruments Inc., Sterling Heights, MI). Discrepant scores between the two investigators were rescored to get a single final score.

### Small interfering RNA-mediated gene silencing of EZH2

EZH2 knockdown in osteosarcoma cells was performed by transfection of synthetic human EZH2 siRNA (AM16708; Ambion at Applied Biosystems, Foster City, USA). The siRNA sequence targeting EZH2 corresponded to coding regions (5′-AGAUCUACAUCGUGAUGAATT-3′, antisense 5′-UUCAUCACGAUGUAGAUCUTG-3′) of the EZH2 gene. The non-specific siRNA oligonucleotides (AM4637; Applied Biosystems) were used as negative controls. 5000 cells per well were seeded in 96-well plates with complete growth medium without antibiotics and transfected with non-specific siRNA or EZH2 siRNA. Transfections were performed with Lipofectamine RNAiMAX (Invitrogen Carlsbad, CA) following the manufacturer's instructions.

### Wound healing and invasion assays

Cell migration activity was assessed by wound healing assay. In brief, cells were seeded onto plates and a scraped; a cellular area was created using a 200 μL tip. We then observed the spread of wound closure and measured the fraction of cell coverage across the line for migration rate. Three images were taken per well at each time point via microscope (Nikon Instruments, Inc.).

Cell invasion activity was evaluated by the Matrigel invasion assay with a BD BioCoat™ Matrigel™ Invasion Chamber (Becton-Dickinson, MA) according to the manufacturer's instructions. Briefly, 1 × 10^4^ cells were seeded into the upper chamber of each well in serum-free medium, and the bottom chambers were filled with medium without antibiotics and containing 10% FBS as a chemoattractant. Transfection reagents were added into the upper chamber. After 48 h, the non-invading cells were gently removed with a cotton swab. Invasive cells located on the lower side of the chamber were stained with crystal violet, air dried, and photographed. For colorimetric assays, the samples were treated with 150 ml 10% acetic acid and the absorbance was measured at 560 nm with a spectrophotometer (Spectramax M5, Molecular Devices, LLC, Sunnyvale, CA).

Images were acquired by Nikon Eclipse Ti-U fluorescence microscope and phase contrast microscope equipped with a SPOT RT digital camera. The number of invading cells was counted in three images per membrane by microscopy using a 20× objective.

### Western blotting

Western blotting reagents and procedures have been previously described [44]. Briefly, total protein was isolated with RIPA Lysis Buffer (Upstate Biotechnology, New York, USA). The concentration of the protein was determined by protein assay reagents (Sigma-Aldrich, St. Louis, MO, USA) with a spectrophotometer (Beckman DU-640, Beckman Instruments, Inc., Indianapolis, IN). 20–30 μg of protein extracted from tumor tissues or cell lysates were separated by SDS-PAGE electrophoresis and then transferred onto nitrocellulose membranes. Membranes were then blocked with 5% non-fat milk in 1× PBS-T and then probed with a specific primary antibody to human EZH2 (1:1000 dilution, Cell Signaling Technology) or mouse monoclonal antibody to human β-actin (Sigma-Aldrich, St. Louis, MO, USA) at 4°C overnight. The membranes were further probed with respective secondary antibodies (LI-COR Biosciences, Lincoln, NE) and scanned by Odyssey^®^ CLx equipment (LI-COR Biosciences) to detect the bands and the density. All other antibodies used in this study were purchased from Santa Cruz Biotechnology or Cell Signaling Technologies. Densitometric analysis of Western blot results was performed with Image J as described in the software's User Guide. The relative expression of protein was normalized to its respective actin expression.

### Proliferation assay

Effects of EZH2 siRNA on cellular growth and proliferation were determined *in vitro* using the MTT assay. 2000 cells were seeded in 96-well plates with complete growth medium without antibiotics in a volume of 100 μL and transfected with non-specific siRNA or EZH2 siRNA. After incubation, 20 μL of MTT (5 mg/mL, Sigma, MO) was added to each well and absorbance was read at a wavelength of 490 nm using a SPECTRAmax Microplate Spectrophotometer (Molecular Devices). All procedures were repeated for five consecutive days.

### Clonogenic assay

Colony formation assay was conducted to investigate the effect of EZH2 on cell growth and proliferation of osteosarcoma cells as described previously. In brief, 48 h after non-specific or EZH2 siRNA transfection, about 1000 single cells were seeded into each well of a 6-well plate. Cells were cultured for 9 to 11 days in the 37°C incubator. Cells were washed by PBS and fixed by 4% paraformaldehyde, stained with 2% crystal violet, and then washed with water. The number of colonies with > 50 cells was counted manually using a microscope.

### Immunofluorescence

To visualize immunofluorescence of EZH2 expression, MG63 and U2OS cells were transfected with non-specific siRNA or EZH2 siRNA in 24-well chambers for 72 h. The samples were fixed with 4% PFA (Sigma-Aldrich) in PBS for 15 min at room temperature, and then permeabilized with ice-cold methanol for 10 min. Unspecific binding of the antibodies was blocked with 1% BSA in PBS-T for 30 min, and then incubated with EZH2 primary antibody (1:50 dilution, Cell Signaling Technology) and β-actin (1:200 dilution, Sigma-Aldrich) at 4°C overnight. Samples were incubated with Alexa Fluor 488 (Green) conjugated goat anti-rabbit antibody (Invitrogen, USA) and Alexa Fluor 594 goat anti-mouse antibody (Invitrogen, USA) for 1 h.

### Three-dimensional cell culture assay

To accurately mimic the environment *in vivo*, a three-dimensional cell culture assay was performed to assess the effect of EZH2 inhibition on the cell growth and proliferation of osteosarcoma cells. Briefly, 48 h after non-specific or EZH2 siRNA transfection, a 5000 cell suspension in a volume of 40 μL was seeded into a Perfecta3D 96-well Hanging Drop plate (3D Biomatrix, USA) following the manufacturer's instructions. Cells were cultured for 7 days in the 37°C incubator and then stained by Calcein AM. Osteosarcoma cell spheres were then visualized and images were acquired with a Nikon Eclipse Ti-U fluorescence microscope (Nikon Instruments) and imaged.

### Flow cytometric analysis of cell apoptosis

The extent of apoptosis was measured with an Annexin V-APC Apoptosis Detection kit (BD Biosciences) according to the manufacturer's instructions. Untransfected or EZH2-siRNA transfected U2OS and SAOS cells were collected, washed with cold PBS twice, gently resuspended in 100 μl of 1× binding buffer containing 2.5 μl APC-conjugated annexin-V and 1 μl of 100 μg/mL PI, and then incubated at room temperature in the dark for 15 min. The stained cells were analyzed by flow cytometry (BD Biosciences). The experiments were performed in triplicate.

### Determination of the effects of the DZNep on the osteosarcoma

DZNep was purchased from Selleck Chemicals (Houston, TX) and resuspended in DMSO. Firstly, we treated the U2OS and SAOS cells with DZNep at different concentrations for 72 h and assessed the effect of DZNep on the expression of EZH2 and proliferation of osteosarcoma cells *in vitro*. At least three independent experiments were performed to determine the half maximal inhibitory concentration (IC50) values for each cell line. IC50 values were determined using GraphPad Prism 4.0 (GraphPad Software, San Diego, CA).

### Statistical analysis

Kaplan-Meier plots and log-rank tests were used for survival analysis. Univariate and multivariate Cox proportional hazard regression models were used to analyze independent prognostic factors. The Student's *t*-test was used to analyze the statistical difference among groups. Data were analyzed by one-way ANOVA followed by Tukey's post hoc test. In all cases, results were presented as mean ± SD and *P* < 0.05 was assumed to be statistically significant. All data points represent the mean of triplicate data points.
